# 

**Published:** 1910-02-19

**Authors:** 


### THE MEDICAL LIBRARY.

| | Post Free. |
|---|---|
| "Medical History: From the Earliest Times." By E. T. Withington, M.A., M.B. Oxon ... | 12s. 6d. |
| "The Consumptive Working Man. What Can the Sanatoria Do for Him?" By Noel D. Bardswell, M.D. ... ... ... ... ... | 10s. 10d. |
| "Photomicrography." By Edmund Spitta, M.R.C.S. With 41 Half-tone Reproductions from original negatives and other Illustrations ... ... | 12s 0d. |
| "Outlines of Insanity." A Popular Treatise on the Salient Features of Insanity. ... ... ... | 1s. 6d. |
| "Sight and Hearing in Childhood." By R. Brudenell Carter, F.R.C.S., and Arthur Cheatle, F.R.C.S. | 2s. 3d. |

Of all booksellers or of The Scientific Press, Limited, 28 & 29 Southampton Street, Strand, London, W.C.

### Gifts and Bequests.

The Royal Victoria Hospital, Belfast, has received £100 from Sir Thomas and Lady Dixon.

The King has sent his annual subscription of ten guineas to the Brompton Hospital for Consumption and Diseases of the Chest.

Mr. Benjamin Cunningham Wates, of Bromley, Kent, has bequeathed £100 to the Leicester Infirmary and House of Recovery from Fever.

The Prince of Wales's General Hospital, Tottenham, has received £100 from the trustees of the Grimthorpe Charity, and £10 10s. from the Leathersellers' Company.

Mr. John Thomson Paton, manufacturer, Alloa, has left £1,000 each to the Royal Infirmary, Edinburgh, the Royal Infirmary, Glasgow, and the Clackmannan County Hospital, Alloa.

Mr. Thomas Skidmore, of Wolverhampton, has left £100 each to the Wolverhampton General Hospital, the Wolverhampton Eye Infirmary, and the Wolverhampton Hospital for Women.

The Treasurer of the Royal Albert Orphan Asylum acknowledges the receipt of a bank-note for £5 from "Excelsior" in response to Lord Northcote's letter which appeared in the *Times* on February 4.

£1,000 has been received by the Royal Free Hospital under the will of the late Mrs. Greenhill to endow a bed, and £400 was received from Mrs. Fernie to endow the "Violet Cot" in memory of the late Dr. Frances Hickman.

Mrs. Caroline Amelia Savage, who left estate of the gross value of £11,506 11s. 11d., and subject to many small bequests, left the residue of her property to the Hospital for Women, Soho Square, W.C. It would appear probable that the sum available for this may amount to £10,000.

The treasurer of Bartholomew's Hospital acknowledges the following further contributions to the Lord Mayor's appeal:—Anonymous, £200; Mr. W. Melville Wills, £50; Mr. W. A. Sturdy, £50; Dr. Jamieson B. Hurry, £25; Mr. W. S. Bowyer, £12 12s.; the Dowager Lady Simeon, £10 10s.; and Major Leonard Darwin, R.E., £10.

The late Mr. Samuel Haldane, a partner in the firm of Pickering, Haldane and Co., Ltd., has left the following bequests:—The Hull Royal Infirmary, £5,000; the Hull and Sculcoates Dispensary, £1,000; the Hull Victoria Hospital for Sick Children, £500; the Hull and East Riding Convalescent Home, Withernsea, £300.

Mr. James Smith, cotton broker, has left £10,000 in trust for religious and charitable institutions in or around Liverpool, £1,000 to the David Lewes Northern Hospital, Liverpool; £1,000 each to the Wallasey Cottage Hospital, Victoria Central Hospital, Liscard; £500 to the New Brighton Convalescent Home and to the Cottage Hospital, Moffat, Dumfries.

Miss Elizabeth Gough, of Long Ashton, Somerset, has left £500 each to the Bristol Royal Infirmary, the Bristol General Hospital, the Bristol Hospital for Sick Children and Women, the West End Hospital for Diseases of the Nervous System, Paralysis, and Epilepsy, London, the Royal Hospital for Incurables, and £200 to the Bristol Dispensary, Castle Green.

Dewsbury Infirmary endowment fund has been augmented by a legacy of £500 bequeathed by the late Miss Ellen Culpan, of Heckmondwike, and £100 by the late Mr. Squire Rayner, of Savile Town; and just recently it has been communicated to the Board that the late Sir Thomas Freeman Firth, Bart., of Heckmondwike, has left to the institution £500. He had long been a warm friend of the infirmary.

### THE QUESTION BOX.

**SPECIAL NOTICE.—Questions of interest affecting the honorary medical staff and hospital administration in all departments are invited. When any point arises we hope our readers will formulate it in a question and so co-operate to make this column of real value and interest. In this way the experience of the best-managed hospitals should be made helpful to the whole hospital world. We shall welcome the contribution of both questions and answers.**

**N.B.—The publication of an answer in this column does not necessarily preclude the publication of subsequent answers to the same question, for a question may involve points which require to be threshed out and fully discussed. Answers, if published, will be paid for at scale rates.**

### A CENTRAL STORE FOR HOSPITALS.

From time to time we have dealt with the question of one central store from which the managers of the various hospitals in combination might secure supplies of the best possible quality. This system, as pursued in France, Belgium, and elsewhere, has been fully described, the matter has been discussed in the hospital world, but in the result no combination has so far taken place. The question has been mooted by some of the more active and younger men in the hospital world, and we therefore invite our readers to discuss it in all its bearings in this column Mr. Frank Debenham took a great interest in the idea some years ago, and in the result the Hospitals and General Contracts Company, Ltd., was incorporated. Although this company has gradually extended the area of its operations, it has not, we believe, attempted to create a central store for the supply of every requisite that each hospital needs. There, of course, the difficulty comes in, for the field is so vast that to be thoroughly well done a large capital and the possession of wide and great practical knowledge on the part of heads of departments are essential to success. We invite our readers to give us their experience in view of the working of the Commissariat Department of their hospital.

The Royal National Pension Fund in a small way has done a great service to its policyholders, being nurses, by establishing an office where each nurse can obtain accurate information and be put in the way of purchasing many things which she may need of the best quality at the smallest cost.

The points to consider in relation to a Central Hospital Store are:—

1. What advantages would each hospital be likely to derive from its establishment?

2. What are the practical objections against its establishment?

3. Is there any intermediate or alternative scheme requiring less capital and organisation which might be tried experimentally (*a*) to help the hospitals to help themselves with regard to certain classes of supplies, and (*b*) if the attempt proved successful, which might lead to the carrying out of the larger scheme?

4. Is it a fact that in practice a well-organised Commissariat Department at a great hospital can purchase the best articles in requisite quantities at a price so low as to render the institution of one Central Hospital Store unnecessary or even undesirable?

The whole idea may usefully be threshed out, and we invite communications on the subject from all who have any practical knowledge upon any of the questions involved. We hope those who accept this invitation will express their views and experiences quite frankly, so that in the result much practical information with regard to hospital supplies may be made available for all who are interested in the economical administration of hospitals of every kind.

THE
### GRADUATES' MEDICAL SCHOOLS.

DIARY FOR THE WEEK, FEBRUARY 21 to 26.

### THE POST-GRADUATE COLLEGE, West London Hospital, Hammersmith, W.

**At 10 a.m.—Feb. 21 and Feb. 24, Demonstration by Surgical Registrar.**
**10 a.m.—Feb. 25, Demonstration by Medical Registrar.**
**12 noon.—Feb. 24,** Dr. Bernstein, **Pathological Demonstration.**
**12.15 p.m.—Feb. 23,** Dr. Grainger Stewart, **Practical Medicine.**
**5 p.m.—Feb. 21,** Mr. Baldwin, **Practical Surgery—** Lecture IV.
**5 p.m.—Feb. 22,** Dr. Pritchard, **Clinical Pathology.**
**5 p.m.—Feb. 23,** Mr. Rickard Lloyd, **Anæsthetics.**
**5 p.m.—Feb. 24,** Dr. Grainger Stewart, **Vascular Diseases of the Brain and their Treatment.**
**5 p.m.—Feb. 25,** Dr. Cole, **General Treatment of Mental Diseases.**

### ROYAL SOCIETY OF MEDICINE, 20 Hanover Square, W.

**At 5.30 p.m.—Feb. 22, Medical Section:**
*Discussion* on "Pericarditis with effusion as determined by operation or P.M. examination," opened by Dr. Samuel West.
Members of the Section for the Study of Disease in Children are specially invited to take part in the discussion.

**At 8.30 p.m.—Feb. 24, Neurological Section:**
*Papers (with Specimens and Epidiascope Illustrations).*—Mr. Hinds Howell: Tumours of the pineal body. Dr. Campbell Thomson: Thrombosis, ? pontine, causing left fifth nerve anæsthesia, and hemianalgesia of the same side. Dr. Wilfred Harris: A case of thrombosis of the posterior inferior cerebellar artery, followed by severe trigeminal neuralgia in the analgesic facial area.

**At 4.30 p.m.—Feb. 25, Section for the Study of Disease in Children:**
*Cases.*—Dr. T. R. Whipham: Myelogeneous leukæmia in an infant of eighteen months. Mr. P. Lockhart Mummery: Congenital separation of the recti abdominis and double inguinal hernia in a girl. Dr. David Forsyth: Non-cretinous idiocy with goitre. Dr. G. Pernet: Lupus vulgaris. Mr. S. Maynard Smith: Congenital œdema of the leg. And other cases.
*Specimens.*—Dr. W. Essex Wynter: Specimens and colour photographs from a case of chloroma, shown at a former meeting. Dr. Walter Carr: Granular kidneys from a boy of twelve.

**At 8.30 p.m.—Feb. 25, Epidemiological Section:**
*Paper.*—Dr. J. E. Sandilands: The communication of diarrhœa from the sick to the healthy.

### Appointments Vacant.

Beckett Hospital and Dispensary, Barnsley.—Hon. Ophthalmic Surgeon.
Liverpool Hahnemann Hospital and Dispensary.—Medical Officer.

THE HOSPITAL. February 19, 1910.

*Name* ..................................................

*Address* ..................................................

..................................................

This Coupon must accompany manuscript or contributions intended for The Hospital.

